# Supplementary material for: Variation in the Floral Morphology of Prosthechea karwinskii (Orchidaceae), a Mexican Endemic Orchid at Risk
Source: Plants (Basel). 2024 Jul 20;13(14):1984. doi: 10.3390/plants13141984 (PMC11280756; doi:10.3390/plants13141984)
Supplement: Supplementary file 1 [file plants-13-01984-s001.zip › plants-3098308-supplementary.pdf]

Table S1: Results of the ANOVA and Tukey tests for the floral characters recorded in 12 expanded populations of *Prosthechea karwinskii* from Oaxaca, including individuals rescued from Zaachila. The values in parentheses show the mean ± standard error (in mm) for each floral variable recorded in each population. The populations did not show significant difference among themselves when the value of the variable shared the same script letter; when the script letter was different, there were significant differences between populations (with p<0.05). See Table 4 for variable names.

Variable	Expanded populations											
	Sola_El Lazo	Jaltianguis	Juquila	Sola_Rancho Viejo	Etla	Albarradas	Sola_Lachixio	Teposcolula	Tlaxiaco	Yahuiche	Yanhuitlan	Zaachila
SlLt	(57.41 ± 2.70)ab	(55.90 ± 1.47)ab	(53.85 ± 1.69)ab	(48.96 ± 1.02)b	(54.92 ± 1.59)ab	(53.50 ± 2.34)b	(55.81 ± 1.60)ab	(61.69 ± 1.85)a	(56.03 ± 2.12)ab	(56.75 ± 1.24)ab	(53.78 ± 1.76)b	(55.28± 1.40)ab
SlAm	(16.84 ± 0.96)ab	(15.97 ± 0.84)ab	(15.51 ± 0.49)ab	(14.10 ± 0.49)b	(15.14 ± 0.52)ab	(15.94 ± 0.71)ab	(15.34 ± 0.60)ab	(16.94 ± 0.67)a	(15.43 ± 0.63)ab	(15.42 ± 0.33)ab	(14.17 ± 0.51)b	(14.77 ± 0.32)ab
SlA1	(15.92 ± 0.91)a	(14.89 ± 0.89)ab	(14.82 ± 0.48)ab	(13.33 ± 0.49)ab	(14.22 ± 0.56)ab	(15.30 ± 0.67)ab	(14.37 ± 0.65)ab	(15.73 ± 0.72)a	(14.29 ± 0.65)ab	(14.39 ± 0.29)ab	(12.87 ± 0.59)ab	(13.70 ± 0.36)ab
SlA2	(15.15 ± 0.91)a	(14.98 ± 0.62)ab	(14.44 ± 0.40)ab	(13.40 ± 0.48)b	(14.92 ± 0.88)ab	(15.00 ± 0.64)ab	(14.34 ± 0.56)ab	(16.18 ± 0.61)ab	(14.82 ± 0.62)ab	(14.37 ± 0.30)ab	(13.05 ± 0.57)b	(14.14 ± 0.33)ab
SlLa	(13.05 ± 0.57)	(13.05 ± 0.57)	(13.05 ± 0.57)	(13.05 ± 0.57)	(13.05 ± 0.57)	(25.60 ± 1.93)	(13.05 ± 0.57)	(13.05 ± 0.57)	(13.05 ± 0.57)	(13.05 ± 0.57)	(13.05 ± 0.57)	(13.05 ± 0.57)
SlAa	(50.83 ± 2.32)	(58.27 ± 2.46)	(61.21 ± 2.68)	(59.23 ± 2.22)	(60.05 ±02.35)	(48.16 ± 4.11)	(57.55 ± 2.44)	(59.69 ± 2.72)	(63.06 ± 2.73)	(61.47 ± 2.15)	(58.95 ± 2.34)	(62.84 ± 1.93)
SdLt	(54.98 ± 1.85)abc	(53.33 ± 1.46)abc	(50.86 ± 1.66)bc	(46.09 ± 1.10)c	(53.30 ± 1.37)abc	(51.50 ± 2.41)abc	(52.71 ± 1.78)abc	(60.35 ± 1.58)a	(53.64 ± 1.32)abc	(55.66 ± 1.32)ab	(51.46 ± 1.65)bc	(53.62 ± 1.33)abc
SdAm	(18.38 ± 1.07)ab	(17.54 ± 0.91)ab	(16.60 ± 0.67)ab	(15.26 ± 0.52)b	(16.48 ± 0.51)ab	(17.50 ± 0.89)ab	(16.71 ± 0.68)ab	(18.42 ± 0.65)a	(16.99 ± 0.56)ab	(16.61 ± 0.33)ab	(15.65 ± 0.57)b	(16.07 ± 0.36)ab
SdA1	(16.78 ± 1.00)	(15.83 ± 0.85)	(15.43 ± 0.74)	(14.27 ± 0.49)	(14.99 ± 0.63)	(16.03 ± 0.85)	(15.41 ± 0.66)	(16.45 ± 0.74)	(14.91 ± 0.71)	(15.16 ± 0.31)	13.87 ± 0.65)	(14.08 ± 0.38)
SdA2	(17.24 ± 0.67)ab	(16.96 ± 0.67)ab	(16.06 ± 0.70)ab	(14.65 ± 0.51)b	(15.92 ± 0.50)ab	(16.56 ± 0.79)ab	(16.11 ± 0.64)ab	(17.97 ± 0.65)a	(16.2 ± 0.58)ab	(16.02 ± 0.36)ab	(14.95 ± 0.60)b	(15.71 ± 0.37)ab
SdLa	(24.63 ± 1.61)	(24.82 ± 0.58)	(26.25 ± 1.73)	(22.21 ± 0.81)	(25.82 ± 1.17)	(23.61 ± 1.84)	(23.08 ± 1.28)	(25.50 ± 1.96)	(24.44 ± 1.42)	(26.16 ± 0.97)	(23.02 ± 1.08)	(22.28 ± 0.82)
SdAa	(55.66 ± 3.76)	(63.09 ± 3.92)	(67.35 ± 2.24)	(65.95 ± 2.13)	(64.36 ± 1.89)	(56.16 ± 5.76)	(67.65 ± 3.54)	(61.61 ± 3.64)	(70.53 ± 3.38)	(68.15 ± 2.39)	(66.54 ± 2.30)	(68.10 ± 2.28)
PeLt	(50.85 ± 2.06)bc	(51.11 ± 1.30)bc	(49.16 ± 1.68)bc	(45.02 ± 0.99)c	(51.50 ± 1.47)abc	(49.41 ± 2.33)bc	(51.21 ± 1.59)bc	(58.71 ± 1.71)a	(52.22 ± 2.07)ab	(53.54 ± 1.32)ab	(49.77 ± 1.53)bc	(51.58 ± 1.26)ab
PeAm	(24.75 ± 2.20)ab	(21.80 ± 1.01)ab	(24.00 ± 0.88)ab	(19.81 ± 0.77)ab	(22.32 ± 0.94)ab	(24.25 ±1.43)ab	(21.81 ± 1.07)ab	(26.25 ± 1.43)a	(23.23 ± 1.11)ab	(23.17 ± 0.67)ab	(21.19 ± 0.85)b	(21.27 ± 0.56)b
PeA1	(18.71 ± 1.51)ab	(18.44 ± 0.87)ab	(21.31 ± 0.76)ab	(17.48 ± 0.79)b	(19.60 ± 0.82)ab	(21.31 ± 1.29)ab	(19.06 ± 1.06)ab	(23.11 ± 1.52)a	(20.17± 1.52)ab	(20.43 ± 0.61)ab	(17.94 ± 0.77)b	(17.74 ± 0.62)b
PeA2	(23.04 ± 1.56)ab	(19.97 ± 0.84)ab	(21.42 ± 0.78)ab	(18.14 ± 0.68)b	(20.09 ± 0.89)ab	(21.51 ± 1.21)ab	(19.55 ± 0.90)ab	(23.36 ± 1.32)a	(20.58 ± 1.10)ab	(20.22 ± 0.54ab)	(18.91 ± 0.84)b	(19.64 ± 0.48)ab
PeLa	(25.51 ± 0.99)ab	(24.73 ± 0.93)ab	(25.23 ± 1.19)ab	(23.17 ± 0.84)b	(26.43 ± 1.08)ab	(24.31 ± 1.22)ab	(25.90 ± 1.10)ab	(29.16 ± 1.44)a	(27.34 ± 1.12)ab	(27.84 ± 0.76)a	(24.77 ± 0.94)ab	(24.20 ± 0.87)ab
PeAa	(67.66 ± 4.82)cd	(77.54 ± 4.23)abcd	(78.78 ± 4.39)abcd	(80.76 ± 2.12)abcd	(86.10 ± 2.85)abc	(60.66 ± 2.51)d	(77.80 ± 3.51)abcd	(73.38 ± 3.81)bcd	(91.33 ± 3.48)a	(81.42 ± 2.45)abc	(87.36 ± 2.95)abc	(88.10 ± 3.06)ab
LaLt	(61.03 ± 2.53)abc	(56.42 ± 0.94)bc	(56.49 ± 1.64)bc	(51.39 ± 1.15)c	(57.75 ± 1.67)abc	(57.31 ± 1.84)abc	(57.82 ± 1.59)abc	(65.51 ± 1.68)a	(62.12 ± 2.12)ab	(56.48 ± 1.62)bc	(56.19 ± 1.73)bc	(58.67 ± 1.35)abc
LaAm	(41.13 ± 1.54)ab	(39.99 ± 1.18)ab	(42.14 ± 1.16)ab	(37.40 ± 1.19)b	(38.95 ± 0.95)b	(42.05 ± 0.99)ab	(40.82 ± 1.50)ab	(44.90 ± 1.20)a	(42.30 ± 1.56)ab	(40.39 ± 0.88)ab	(38.71 ± 1.16)b	(40.70 ± 1.01)ab
LaLl	(35.00 ± 2.15)abc	(34.36 ± 1.33)abc	(35.66 ± 1.46)abc	(31.76 ± 0.80)c	(36.05 ± 0.97)abc	(36.06 ± 1.71)abc	(36.07 ± 1.28)abc	(39.28 ± 1.31)a	(37.52 ± 1.15)bc	(33.74 ± 0.81)bc	(33.99 ± 1.06)bc	(36.14 ± 0.81)abc
LaAml	(11.63 ± 0.75)abc	(10.71 ± 0.66)bc	(12.29 ± 0.53)abc	(10.42 ± 0.44)c	(10.19 ± 0.35)c	(14.05 ± 0.35)a	(10.72 ± 0.46)bc	(12.69 ± 0.33)ab	(12.87 ± 0.68)ab	(11.48 ± 0.43)abc	(11.09 ± 0.44)abc	(10.91 ± 0.84)bc

LaA1l	(11.42 ± 0.49)abc	(9.61 ± 0.62)abc	(11.51 ± 0.66)abc	(9.50 ± 0.45)bc	(9.20 ± 0.35)c	(12.75 ± 0.19)a	(9.68 ± 0.44)abc	(11.62 ± 0.44)ab	(11.73 ± 0.71)ab	(10.61 ± 0.44)abc	(9.96 ± 0.45)abc	(9.92 ± 0.41)abc
LaAul	(137.00 ± 3.35)abcd	(137.54 ± 4.36)abc	(130.64 ± 3.18)abcd	(139.23 ± 2.32)ab	(121.94 ±1.84)d	(96.16 ± 1.62)e	(131.30 ± 3.94)abcd	(126.76±3.29)bcd	(123.73±3.29)cd	(141.52±1.72)a	(131.13±2.39)abcd	(125.78±3.32)cd
LaAlm	(107.16±11.53)	(110.81 ± 7.58)	(99.14 ± 7.26)	(106.66 ± 4.02)	(118.79 ±4.01)	(86.66 ± 1.66)	(109.55 ± 4.57)	(113.92±4.24)	(110.86±5.98)	(105.73±4.68)	(112.81±4.92)	(105.84 ±5.04)
LaLm	(18.86 ± 1.34)ab	(16.53 ± 0.51)abc	(15.73 ± 0.40)bc	(14.42 ± 0.45)c	(18.66 ± 0.63)ab	(17.15 ± 0.88)abc	(17.57 ± 0.67)ab	(20.03 ± 0.78)a	(17.35 ± 0.54)abc	(18.40 ± 0.59)ab	(17.07 ± 0.76)abc	(17.45 ± 1.77)ab
LaAbm	(19.43 ± 0.64)	(19.54 ± 0.70)	(18.27 ± 0.71)	(17.97 ± 0.56)	(20.21 ± 0.65)	(18.82 ± 0.56)	(19.90 ± 0.77)	(20.58 ± 1.14)	(19.13 ± 0.65)	(19.91 ± 0.69)	(17.54 ± 0.75)	(19.54 ± 0.48)
LaAmm	(20.27 ± 0.59)ab	(20.14 ± 0.60)ab	(19.35 ± 0.82)ab	(18.72 ± 0.58)ab	(20.42 ± 0.63)ab	(19.52 ± 0.59)ab	(20.28 ± 0.79)ab	(21.51 ± 1.03)a	(19.56 ± 0.60)ab	(20.26 ± 0.70)ab	(17.82 ± 0.75)b	(19.77 ± 0.50)ab
LaAam	(156.8.78± 0.59)	(144.782 ± 0.59)	(161.14 ±6.73)	(154.66 ± 5.63)	(161.05 ± 5.88)	(156.16 ± 5.57)	(162.95 ± 5.40)	(135.84 ± 5.40)	(144.66 ± 6.53)	(158.00 ± 6.34)	(162.22 ± 5.36)	(157.42 ± 9.09)
CoLt	(27.77 ± 0.74)ab	(24.47 ± 0.39)c	(27.13 ± 0.33)b	(25.69 ± 0.30)bc	(26.16 ± 0.40)bc	(25.98 ± 1.27)bc	(26.32 ± 0.51)bc	(29.69 ± 0.51)a	(27.55 ± 0.62)ab	(26.05 ± 0.41)bc	(25.39 ± 0.54)bc	(26.91 ± 0.38)bc
CoAe	(9.31 ± 0.46)	(8.34 ± 0.29)	(8.72 ± 0.30)	(8.35 ± 0.20)	(8.52± 0.34)	(8.34 ± 0.56)	(8.94 ± 0.29)	(9.17 ± 0.31)	(9.12 ± 0.33)	(8.69 ± 0.14)	(7.88 ± 0.35)	(7.80 ± 0.34)
CoA1	(6.73 ± 0.43)ab	(7.11 ± 0.28)a	(6.53 ± 0.31)ab	(6.19 ± 0.13)ab	(7.08 ± 0.28)a	(5.87 ± 0.49)ab	(6.52 ± 0.32)ab	(6.86 ± 0.28)ab	(6.82 ± 0.37)ab	(6.97 ± 0.14)a	(5.89 ± 0.38)ab	(5.58 ± 0.24)b
CoGm	(7.23 ± 0.46)	(6.84 ± 0.26)	(6.61 ± 0.34)	(6.30 ± 0.16)	(6.98 ± 0.31)	(5.30 ± 0.30)	(6.58 ± 0.30)	(7.32 ± 0.32)	(7.18 ± 0.38)	(6.93 ± 0.15)	(5.65 ± 0.41)	(5.62 ± 0.20)
CoGa	(9.08 ± 0.31)	(8.46 ± 0.20)	(7.98 ± 0.33)	(8.26 ± 0.15)	(8.21 ± 0.30)	(7.44 ± 0.26)	(8.42 ± 0.30)	(9.08 ± 0.22)	(9.01 ± 0.33)	(8.75 ± 0.14)	(7.60 ± 0.37)	(7.44 ± 0.17)
RoAl	(8.29 ± 0.50)a	(6.45 ± 0.26)ab	(7.90 ± 0.30)a	(7.56 ± 0.26)ab	(7.09 ± 0.18)ab	(6.86 ± 0.36)ab	(7.93 ± 0.22)a	(7.01± 0.44)ab	(7.49± 0.25)ab	(7.34 ± 0.20)ab	(7.14 ± 0.29)ab	(6.39 ± 0.52)b
RoAn	(7.88 ± 0.36)a	(6.38 ± 0.26)ab	(7.26 ± 0.33)ab	(6.84 ± 0.27)ab	(7.08 ± 0.32)ab	(6.40 ± 0.66)ab	(7.42 ± 0.31)a	(6.91 ± 0.47)ab	(7.26 ± 0.39)ab	(6.89 ± 0.31)ab	(6.41 ± 0.31)ab	(5.94 ± 0.30)ab
DlAl	(5.10 ± 0.32)bc	(3.71 ± 0.11)de	(5.69 ± 0.28)ab	(4.33 ± 0.13)cd	(5.22 ± 0.14)b	(2.96 ± 0.52)e	(4.85 ± 0.16)bc	(6.20 ± 0.24)a	(5.43 ± 0.27)ab	(4.17 ± 0.09)cde	(4.88 ± 0.17)bc	(5.30 ± 0.16)ab
DlAn	(4.94 ± 0.20)ab	(5.10 ± 0.10)ab	(4.66 ± 0.25)ab	(5.04 ± 0.19)ab	(4.51 ± 0.17)b	(4.05 ± 0.44)b	(5.29 ± 0.16)ab	(5.70 ± 0.23)a	(5.35 ± 0.27)ab	(5.55 ± 0.11)a	(4.90 ± 0.26)ab	(4.90 ± 0.26)ab
DmAl	(4.00 ± 0.13)de	(4.37 ± 0.18)cd	(4.00 ± 0.30)de	(4.71 ± 0.18)cd	(5.90 ± 0.15)ab	(2.70 ± 0.15)e	(4.09 ± 0.16)d	(4.26 ± 0.16)d	(4.74 ± 0.19)cd	(4.85 ± 0.08)cd	(5.18 ± 0.17)bc	(6.01 ± 0.16)a
DmAn	(3.74 ± 0.22)	(2.90 ± 0.12)	(2.91 ± 0.07)	(3.25 ± 0.14)	(3.60 ± 0.22)	(2.88 ± 0.13)	(3.68 ± 0.24)	(3.15 ± 0.16)	(3.46 ± 0.15)	(3.40 ± 0.14)	(3.35 ± 0.15)	(3.37 ± 0.11)
AnDlDm	(1.36 ± 0.07)abcd	(1.29 ± 0.08)bcd	(1.11 ± 0.13)bcd	(1.15 ± 0.10)bcd	(1.49 ± 0.10)abc	(2.11 ± 0.41)a	(1.32 ± 0.09)bcd	(1.02 ± 0.11)cd	(0.92 ± 0.10)d	(1.52 ± 0.07)ab	(1.00 ± 0.06)d	(1.00 ± 0.101)d
CuLt	(14.58 ± 0.64)a	(10.90 ± 0.48)b	(11.62 ± 0.53)ab	(11.54 ± 0.42)ab	(12.83 ± 0.50)ab	(12.69 ± 0.11)ab	(13.03 ± 0.58)ab	(12.74 ± 0.66)ab	(13.10 ± 0.62)ab	(11.55 ± 0.49)ab	(12.40 ± 0.42)ab	(12.70 ± 0.49)ab
CuAm	(3.62 ± 0.14)a	(2.70 ± 0.24)ab	(2.84 ± 0.15)ab	(2.74 ± 0.14)ab	(2.96 ± 0.10)ab	(3.47 ± 0.06)a	(2.74 ± 0.14)ab	(2.85 ± 0.17)ab	(3.16 ± 0.12)ab	(2.51 ± 0.12)b	(3.06 ± 0.12)ab	(3.14 ± 0.14)ab
CuA1	(2.02 ± 0.14)ab	(1.81 ± 0.15)ab	(1.74 ± 0.17)ab	(1.53 ± 0.10)ab	(2.05 ± 0.12)a	(2.22 ± 0.13)a	(1.72 ± 0.12)ab	(1.79 ± 0.08)ab	(2.06 ± 0.15)a	(1.43 ± 0.05)b	(1.80 ± 0.11)ab	(2.04 ± 0.09)a
CuA2	(2.85 ± 0.08)a	(2.36 ± 0.16)ab	(2.26 ± 0.18)ab	(2.12 ± 0.12)ab	(2.51 ± 0.11)ab	(2.73 ± 0.12)ab	(2.29 ± 0.13)ab	(2.32 ± 0.14)ab	(2.50 ± 0.16)ab	(1.96 ± 0.11)b	(2.19 ± 0.12)ab	(2.43 ± 0.12)ab
